# Effect of Dietary Urea in Gestating Beef Cows: Circulating Metabolites, Morphometrics, and Mammary Secretions

**DOI:** 10.3390/ani13010006

**Published:** 2022-12-20

**Authors:** Ligia D. Prezotto, Jennifer F. Thorson

**Affiliations:** 1 Department of Animal Science, University of Nebraska—Lincoln, 3940 Fair St., Lincoln, NE 68583-0908, USA; 2 USDA, Agricultural Research Service, U.S. Meat Animal Research Center, Clay Center, NE 68933-0166, USA

**Keywords:** bovine, gestation, lactation, metabolism, nutrition, urea

## Abstract

**Simple Summary:**

Feed costs represent 70 to 90% of total costs in beef cattle systems, with protein being the most expensive component. One of the most cost-effective means to supply protein precursor to ruminants is through dietary urea supplementation. Therefore, we set out to evaluate the influence of urea supplementation during gestation on circulating concentrations of maternal metabolites, body measures, and mammary secretion composition. In this work, we have demonstrated that the pregnant beef cow undergoes metabolic adaptation to maintain pregnancy. However, urea supplementation failed to improve any of the body or mammary parameters assessed. Therefore, it is imperative that novel supplementation strategies be developed for beef cows that maintain the body mass of the dam and improve mammary secretion quantity and quality to ultimately improve the health and productivity of both the cow and calf.

**Abstract:**

Prolific use of supplementation strategies, including the utilization of urea, are practiced in beef cattle production systems. Unfortunately, the influence of urea supplementation on metabolics, adipose tissue mobilization, and mammary secretions is limited in beef cows. Therefore, the objectives of this experiment were to assess the influence of urea supplementation on metabolic profiles, morphometrics, and mammary secretions. Pregnant, multiparous beef cows were fed individually and assigned to treatment (n = 4/treatment) as Control or Urea Supplementation. Blood samples and body weight were collected every 28 d throughout gestation. Backfat thickness was measured via ultrasonography on days 28 and 280 of gestation. Total mammary secretions were sampled for composition. Concentrations of beta-hydroxybutyrate, non-esterified fatty acids, glucose, and plasma urea nitrogen did not differ by treatment. Body weight and backfat thickness changed in response to the progression of gestation, but did not differ between treatments. Finally, concentration of urea nitrogen increased in mammary secretions of cows fed urea, but total content of urea nitrogen in mammary secretions did not differ between treatments. In conclusion, we have demonstrated that the pregnant beef cow undergoes metabolic adaptation during gestation. However, urea supplementation failed to improve any of the morphometric parameters of the dams assessed.

## 1. Introduction

Feed costs represent 70 to 90% of total costs in beef cattle systems, with protein being the most expensive component. Approximately 74.7% of US producers offer some type of dietary protein supplementation strategy to gestating cows [1]. One of the most cost-effective means to supply protein precursor to ruminants is through dietary urea supplementation. As a result of the prolific utilization of protein supplementation in production and less expensive means to offer nitrogen to ruminants using urea, it is critical to thoroughly assess maternal metabolic adaptation to dietary supply on parameters that directly influence the health and productivity of the calf and recrudescence of reproductive cyclicity of the cow.

Circulating metabolites, such as beta-hydroxybutyrate (BHB), non-esterified fatty acids (NEFA), glucose, and plasma urea nitrogen (PUN) are indicators of energetic status and provide insight into nutrient partitioning between cow and fetus during gestation [2]. Most tissues require glucose as the primary source of energy. However, during nutritional stress, an increase in circulating concentration of BHB, which is a product of incomplete fatty acid oxidation is observed [3]. Moreover, during periods of reduced energy supply, adipose tissue is catabolized, resulting in an increased concentration of non-esterified fatty acids (NEFA) that can be converted to glucose by the liver [4]. Alternatively, protein can be utilized as a source of glucose precursor that can be detected by an increase in circulating concentrations of PUN [5].

Synthesis of mammary secretions is initiated during the last 4 to 6 wk of gestation, which increases the energetic demands of the dam. As mammary secretions are generated, the mobilization of energetic stores and altered utilization of nutrients becomes necessary [5]. A 6% increase in dietary crude protein during lactation promotes increased dry matter intake and milk production [6] accompanied by reduced circulating concentration of BHB while concentrations of NEFA remain unaltered.

By characterizing the influence of maternal supplementation of urea on circulating metabolites throughout gestation, it will be possible to improve supplementation strategies and better understand maternal-fetal nutrient partitioning. Literature on the influence of maternal nutrition on circulating concentrations of metabolites during gestation is limited. Therefore, the objectives of this study were to (1) characterize circulating concentrations of metabolites and maternal morphometrics in response to the progression of gestation and (2) evaluate the influence of urea supplementation during gestation on circulating concentrations of metabolites, maternal morphometrics, and quantity and composition of mammary secretions. We hypothesized that the inclusion of dietary urea would result in less mobilization of maternal body reserves and increase the quantity and quality of mammary secretions.

## 2. Materials and Methods

Experiments were conducted in accordance with the Guide for the Care and Use of Agricultural Animals in Agricultural Research and Teaching [7] and were approved by the Montana State University Agricultural Animal Care and Use Committee.

### 2.1. Animals, Experimental Design, and Diets

Multiparous cows (n = 8; 3.4 ± 0.02 yr; 617 ± 90 kg at onset of experiment) utilized in this experiment were the product of Angus cows that were paternal half-siblings bred by one line-bred Hereford sire in a 30-d breeding season to reduce the influence of genetic variation, age, and environment on parameters of interest. At onset of the experiment, cows were non-lactating and at least 120-d postpartum. Cows were housed in a drylot fitted with a Calan Broadbent Feeding System (American Calan; Northwood, NH, USA) and acclimated to a base diet for 28 d. The base diet consisted of chopped hay and wheat straw formulated to meet or exceed dietary recommendations [8]. During the acclimation period, animals were allowed access to all gates and had *ad libitum* access to water and trace mineralized salt. Following acclimation, animals were assigned gates and thereafter feed was offered individually in a single meal fed at 1200 h. Animals had *ad libitum* access to water for the duration of the experiment. Orts were recorded on a daily basis in order to calculate daily feed intake.

Estrus was synchronized using a Select Synch + CIDR^®^ protocol. At the end of the protocol, estrus behavior was monitored every 12 h for 72 h by a single, trained technician. Twelve hours after first observed estrus behavior, artificial insemination was performed. All animals that failed to express estrus behavior during the 72 h immediately following prostaglandin (25 mg; IM) administration were administered GnRH (100 µg; IM) and artificially inseminated. Semen utilized for artificial insemination was collected from one of two related Angus sires. Pregnancy was confirmed by transrectal ultrasonography 28 d after insemination.

After pregnancy was confirmed (day 28), animals were assigned randomly to dietary treatment by BW and backfat thickness: **Control** (0 g urea/animal/day) or **Urea** (80 g urea/animal/day). The Control and Urea diets were isocaloric and consisted of chopped hay and wheat straw top-dressed with a vitamin and mineral pellet to meet or exceed dietary recommendations [8]. The vitamin and mineral pellet offered to the Urea treatment served as the vehicle to deliver dietary urea. Samples of all ingredients were collected throughout the study and stored at −20 °C. Analyses of ingredients were performed by a commercial laboratory (Midwest Laboratory; Omaha, NE, USA) to determine chemical composition (Table 1).

### 2.2. Metabolites

Once every 28 d throughout gestation, blood was collected via jugular venipuncture from cows that had feed withheld for 16 h. If parturition occurred before 280 d of gestation, blood was collected on the day of parturition or the day following parturition to ensure a 16-h feed withdrawal. Blood samples were collected in evacuated tubes containing EDTA for the collection of plasma. Tubes were placed immediately on ice for 60 min. Blood samples were centrifuged at 2000× *g* for 15 min at 4 °C, plasma harvested, and plasma stored at −20 °C until analysis. Plasma samples were analyzed using commercially available ELISA kits for determination of BHB (Sigma-Aldrich, St. Louis, MO, USA; MAK041), NEFA (Zenbio, Durham, NC, USA; SFA-5), glucose (Thermo Fisher Scientific, Waltham, MA, USA; TR15421), and PUN (Invitrogen, Waltham, MA, USA; EIABUN).

### 2.3. Morphometrics

Every 28 d throughout gestation, BW was collected from cows that had feed withheld for 16 h. Diets were adjusted every 28 d for maintenance of 0.113 kg BW gain/day over the course of the experiment and the progression of gestation.

Ultrasonography was utilized to assess backfat thickness on days 28 and 280 of gestation. If parturition occurred before 280 d of gestation, backfat thickness was collected on the day of parturition. At collection, animals were immobilized in a stanchion, hair was clipped over collection area, and mineral oil was applied to permit acoustic coupling between the transducer and animal. Measurements were taken between and parallel to the 12th and 13th rib with the ultrasonography transducer positioned over the longissimus dorsi muscle. Images were collected on the left side of each animal by a single, trained individual using a real-time SonoSite Edge II imaging system (SonoSite, Inc.; Bothell, WA, USA) equipped with a 15-cm, 5 to 10 MHz linear array transducer at 7.5 MHz frequency without the use of a stand-off pad. Three measurements of backfat thickness were determined for each animal on each collection day. Images were captured at a depth of 4.2 cm and backfat thickness was measured using the caliper function of the ultrasonography system.

### 2.4. Mammary Secretions

At parturition, calves were permanently removed from the dam prior to nursing. Cows were immobilized in a stanchion, oxytocin (20 units) administered intravenously, and mammary glands completely evacuated. Mammary secretions were collected within one hour of parturition (Hour 0) and 24 h after the Hour 0 collection (Hour 24). Mammary secretion total volume and weight were recorded and samples collected. Samples collected at Hour 0 were diluted 1:1 with distilled water, while samples collected at Hour 24 were undiluted.

Samples were analyzed by Dairy One Laboratory (Ithaca, NY, USA) for content of fat, lactose, protein, solids-not-fat, somatic cell count, and urea nitrogen. Immunoglobulin (Ig) content was estimated in mammary secretions at 21 °C collected at Hour 0 using a colostrometer. Dilution factor was standardized prior to calculating total content of fat, Ig, lactose, protein, solids-not-fat, somatic cell count, and urea nitrogen in mammary secretions. Total mammary secretion content was calculated as parameter multiplied by mammary secretion weight (fat, lactose, protein, and solids-not-fat) or volume (Ig, somatic cell count, and urea nitrogen).

### 2.5. Statistical Analyses

The effect of maternal dietary treatment on concentrations of circulating metabolites, morphometrics, and mammary secretion quantity and composition were evaluated by ANOVA, following the Shapiro-Wilk test of normal distribution. The sources of variation were treatment, time, and their interaction. The least squares means procedure was used to compare means if significant differences were detected. Data are reported as least squares means ± SEM, unless stated otherwise. Significance was declared at *p ≤* 0.05.

## 3. Results

### 3.1. Metabolites

**Figure 1:**
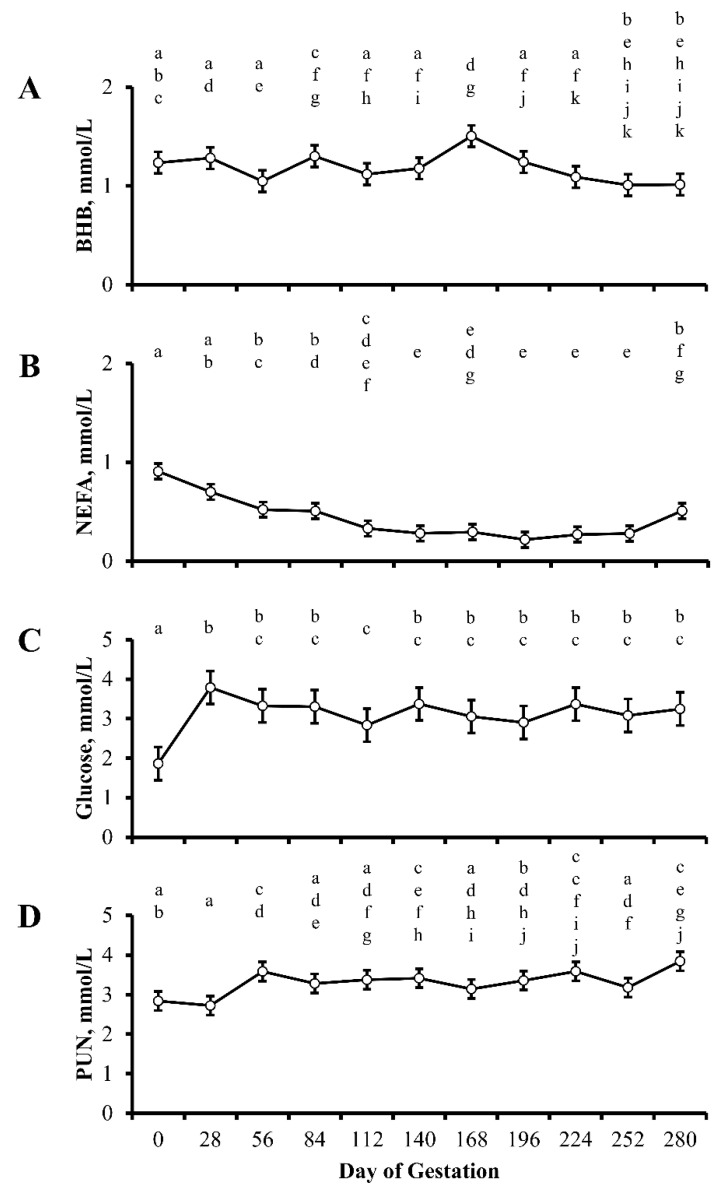
Least squares means ± SEM (mmol/L) of beta-hydroxybutyrate (BHB; **A**), NEFA (**B**), glucose (**C**), and plasma urea nitrogen (PUN; **D**) in multiparous cows by day of gestation. Dietary treatments were: Control (0 g urea/animal/day) or Urea (80 g urea/animal/day). Treatments were offered from day 28 of gestation to parturition. Concentrations of BHB, NEFA, glucose, and PUN did not differ by treatment (*p* ≥ 0.06) or treatment by day interaction (*p* ≥ 0.08), but were influenced by day of gestation (*p* ≤ 0.04) ^a–k^ Days with different lower case letters are different (*p* ≤ 0.05).

Concentrations of BHB, NEFA, glucose, and PUN in cows by day of gestation are illustrated in Figure 1. Concentrations of BHB, NEFA, glucose, and PUN did not differ by treatment (*p* ≥ 0.06) or treatment by day interaction (*p* ≥ 0.08), but were influenced by day of gestation (*p* ≤ 0.04; Table 2). Concentrations of BHB throughout gestation did not differ (*p* = 0.06) from day 0, excluding an increase (*p* = 0.03) in concentrations of BHB observed at 168 d of gestation (Figure 1A). Concentrations of NEFA (Day *p* < 0.0001) decreased (*p* < 0.0001) from day 0 to day 112 of gestation and remained (*p* ≥ 0.33) at this reduced concentration through 252 days of gestation (Figure 1B). Concentrations of NEFA increased (*p* = 0.05) between 252 and 280 d of gestation; however, concentrations remained lower (*p* = 0.0009) than concentrations of NEFA observed on day 0. Concentrations of glucose (Day *p* = 0.04) increased (*p* = 0.0001) from day 0 to day 28 of gestation and remained elevated for the remainder of gestation. A single decline (*p* = 0.05) in concentrations of glucose was observed in samples collected at 112 d of gestation (Figure 1C); however, concentrations remained greater (*p* = 0.05) than concentrations of glucose on day 0 of gestation. Finally, concentrations of PUN (Day *p* = 0.01) increased (*p* ≤ 0.05) at 56, 140, 224, and 280 d of gestation, relative to day 0 (Figure 1D).

### 3.2. Morphometrics

**Figure 2:**
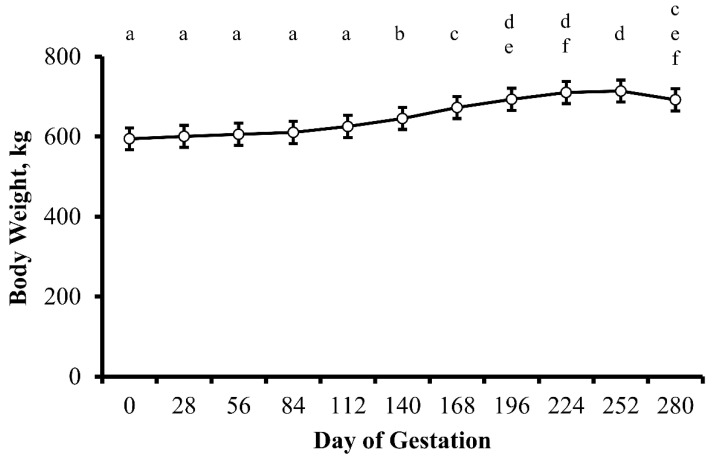
Body weight (kg; least squares means ± SEM) of multiparous cows by day of gestation. Dietary treatments were: Control (0 g urea/animal/day) or Urea (80 g urea/animal/day). Treatments were offered from day 28 of gestation to parturition. Body weight did not change in response to treatment (*p* = 0.75) or treatment by day interaction (*p* = 0.39). However, BW changed (*p* = 0.002) in response to the progression of gestation. ^a-f^ Days with different lower case letters are different (*p* ≤ 0.04).

**Figure 3:**
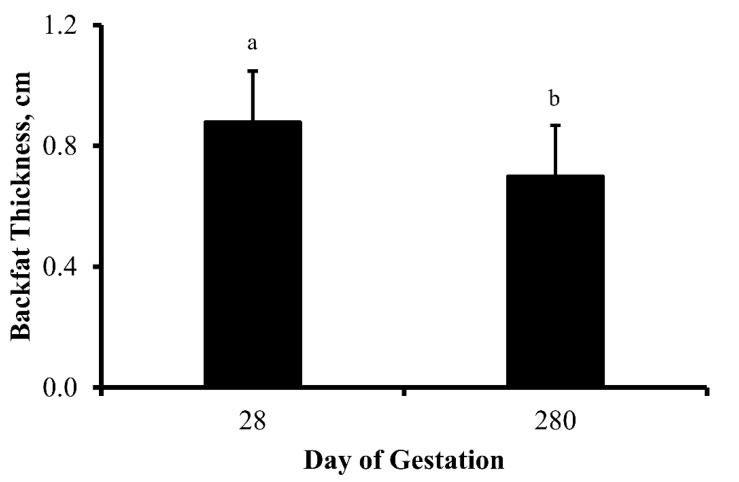
Backfat thickness (cm; least square means + SEM) of multiparous cows on day 28 and 280 of gestation. Dietary treatments were: Control (0 g urea/animal/day) or Urea (80 g urea/animal/day). Treatments were offered from day 28 of gestation to parturition. Backfat thickness did not change in response to treatment (*p* = 0.74) or treatment by day interaction (*p* = 0.65). However, backfat thickness decreased (*p* = 0.02) in response to the progression of gestation. ^a,b^ Days with different lower case letters are different (*p* ≤ 0.02).

There was no influence of treatment (*p* = 0.75) or treatment by day interaction (*p* = 0.39) on cow BW throughout gestation. As predicted, cow BW increased (*p* < 0.0001) as pregnancy progressed from 112 to 252 d of gestation (Figure 2). As parturition neared, cows were unable to consume enough forage to maintain BW resulting in a reduction (*p* = 0.03) in BW between 252 and 280 d of gestation. Moreover, thickness of subcutaneous fat over the ribs (backfat thickness) decreased (*p* = 0.02) between 28 to 280 d of gestation (Figure 3)—illustrating maternal mobilization of energy stores during gestation.

### 3.3. Mammary Secretions

The effects of dietary urea during gestation on mammary secretion volume, weight, and composition are presented in Table 3. There were no differences observed in quantity or quality of the mammary secretions in response to treatment by sample interaction (*p* ≥ 0.18). Concentration of urea nitrogen increased (*p* = 0.02) in mammary secretions of cows fed urea, but total content of urea nitrogen in mammary secretions did not differ (*p* = 0.72) between treatments. Regardless of treatment, the volume and weight of mammary secretions increased (*p* < 0.0001) from Hour 0 to Hour 24. While concentration of lactose did not differ (*p* = 0.11) by sample, total lactose yield was greater (*p* = 0.01) at Hour 24. Concentration of protein and urea nitrogen decreased (*p* = 0.05) from Hour 0 to Hour 24; however, total content of protein and urea nitrogen in mammary secretions did not differ (*p* ≥ 0.64) by sample.

## 4. Discussion

The present study aimed to characterize circulating concentrations of metabolites and maternal morphometrics in response to the progression of gestation and evaluate the influence of urea supplementation during gestation on circulating concentrations of metabolites, maternal morphometrics, and quantity and composition of mammary secretions. The current research illustrates a metabolic shift in dams offered a diet consisting primarily of forage in response to the progression of gestation, a shift that results in the mobilization of maternal adipose tissue to support fetal development. Remarkably, supplemental dietary urea offered to dams was unable to circumvent the mobilization of maternal body reserves or improve the yield of mammary secretions. The authors acknowledge that a possible limitation of the present study is the limited number of animals tested. To fully understand how supplementation and gestation affects metabolic changes in the cow, a larger number of animals with greater genetic variation may be necessary to solely determine what metabolic changes are related to gestation, management, and/or genetic composition in a production setting.

Assessing circulating concentrations of metabolites is a precise tool to measure metabolic and physiological changes in animals in response to physiological state and dietary supply. Abdullah et al. [2] determined that metabolites during early gestation are indicators of energetic balance. Moreover, metabolites can be used to evaluate the metabolic status of the animal prior to breeding, such as BHB that is utilized as a nutritional index of reproductive status of cows [9]. However, no previous reports have assessed the profiles of BHB, NEFA, glucose, and PUN throughout gestation in beef cattle.

Negative energy balance results when energy expenditure exceeds dietary intake. The production of ketone bodies, primarily BHB in ruminants, ensues when mammals enter negative energy balance. Utilization of BHB is a normal process in ruminants through the conversion of butyric acid to BHB in ruminal epithelium before entering the circulation. Once BHB enters the circulation it can be utilized as an alternate energy source by tissues. An increase in circulating concentration of BHB accompanied by a reduction in NEFA can be indicative of a shift towards utilization of alternative energy precursors to maintain homeostasis. This shift can occur during periods of increasing nutritional demand, such as during rapid development of the fetal-placental unit.

In the current experiment, the authors revealed a single increase in circulating concentrations of BHB on day 168 of gestation in beef cattle. Literature regarding BHB in the gestating beef cow is limited. The current report is the first to assess BHB throughout gestation. Wood et al. [10] observed an increase in circulating concentrations of BHB as beef cows transitioned from mid- to late-gestation. Interestingly, these beef cows were able to maintain backfat thickness, potentially as a result of increased feed intake, as diets were offered *ad libitum*. This is in contrast to the current study where feed was limited to meet 100% of nutrient recommendations and maintained isocaloric between treatments. Similar to the current report, Linden et al. [11] demonstrated that concentrations of BHB do not differ during late-gestation (7-wk prepartum). Noya et al. [12] reported concentrations of BHB in beef cows group-fed during early gestation (first 84 d of gestation); however, the influence of progression of gestation on concentration of BHB was not reported.

To our knowledge, the current report is the first to assess BHB in response to urea supplementation in pregnant beef cows. Greenfield et al. [13] demonstrated that inclusion of dietary urea to dairy cows during late gestation (last 28 d of gestation) does not influence circulating concentrations of BHB, similar to what was observed in the current experiment. Eitam et al. [14] reported an increase in circulating concentrations of BHB when protein is restricted in beef cows during mid gestation. While there was no influence of dietary treatment on concentration of BHB in the current experiment, we did observe an influence of day of gestation on BHB and increase in circulating concentrations of BHB on day 168 of gestation—one potentially attributed to fetal secondary myogenesis [15].

In addition to BHB, elevated circulating concentrations of NEFA can also be indicative of negative energy balance in ruminants. Negative energy balance in beef cattle grazing dormant pasture or offered harvested feed can result from increased energy demands during late gestation and the transition to lactation, resulting in reduced BW. Mobilization of fatty acids from adipose tissue occurs mainly in the form of NEFA [16]. Concentrations of NEFA increase in dairy and beef cows as fat is mobilized during the transition period [17,18]. Similarly, the current experiment also demonstrated an increase in circulating concentration of NEFA in beef cows during the periparturient period, irrespective of treatment. Therefore, supplementation of urea greater than dietary recommendations was not effective in alleviating the pregnancy-induced metabolic burden of the dam.

In contrast to the catabolic state of late gestation, early and mid gestation are anabolic in nature for the dam. Factors such as lipoprotein lipase and leptin regulate the uptake of fatty acids from the circulation and adipose metabolic function [19,20,21]. As a result, adipose reserves are increased during early and mid gestation to be utilized during periods of increased nutritional demands. In the current dataset, a decline in circulating concentrations of NEFA in beef cows during early- and mid-gestation was observed—a phenomenon not presented to date. A similar profile occurs during early lactation; but is much shorter in duration with concentrations of NEFA returning to levels observed prior to parturition within 42 d of calving [22]. Cows utilized in the current experiment were at least 120-d postpartum at breeding. Therefore, the authors do not attribute the reduction in circulating concentrations of NEFA observed in the current dataset during early gestation to prior lactation. Instead, the authors propose this decline in concentrations of NEFA during early and mid gestation is a result of both metabolic adaptation of the cow to build adipose depots and reduced fat mobilization. A similar phenomenon occurs in rodents that results in a shift from an anabolic state in early gestation to a catabolic state in late gestation [19,20]. Additional research is needed to explore the role of endocrine factors on maternal adipose reserves in the beef cow during gestation, but it is beyond the scope of the current manuscript.

Glucose synthesized or consumed by the dam serves as the primary fuel for many maternal and fetal tissues [23,24]. Glucose is transported through the placenta via facilitated diffusion down its concentration gradient. Limiting maternal glucose reduces glucose available to the fetus [25], potentially compromising the pregnancy or fetal development. Limited data exists evaluating the effect of dietary urea supplementation on circulating concentrations of glucose in gestating ruminants.

Long et al. [26] investigated the effects of nutrient restriction and demonstrated changes in glucose concentrations. George et al. [27] showed that maternal nutrition during gestation affects the ability of the liver to perform gluconeogenesis and supply the necessary nutrients to the progeny, thus limiting the energy available to maternal tissues. Increased gluconeogenesis in the liver is accompanied by increased mobilization of protein reserves from muscle tissue and consequently affects maternal and fetal substrate depots [28]. However, in the present study, cows supplemented with Urea were not able to maintain greater adipose stores or BW when compared to Control. Moreover, in the current report, circulating concentrations of glucose did not differ between nutritional treatments. Prior reports indicate that the plane of nutrition and macronutrient composition of the diet affects circulating concentrations of glucose [17,29]. Unfortunately, the response to inclusion of urea into the diet varies between reports. Others have indicated that inclusion of dietary urea supplementation decreased [30] or tended to increase [31] circulating concentrations of glucose in lambs. While, in cows, modifying dietary protein via supplementation of urea has been shown to influence circulating concentrations of glucose [29].

In addition to evaluating the influence of dietary urea supplementation on maternal circulating metabolites, we also assessed the influence of progressing gestation. Interestingly in the current report, circulating concentrations of glucose are maintained at a greater concentration after maternal recognition of pregnancy (day 28 to 280) compared to prior to maternal recognition of pregnancy (day 0). Placental lactogen, originating from trophoblast binucleate cells at approximately day 20 of gestation [32], increases circulating concentrations of glucose in the dam [33]. Placental lactogen is detectable in the circulation of pregnant cows as early as day 26 of pregnancy [34] and decreases to undetectable levels within a few days of parturition [35]. This time frame coincides with the elevation in circulating concentrations of glucose in cows in the current dataset. The increase in glucose observed is in alignment with previous studies demonstrating changes in concentrations of glucose during early gestation [2] and the periparturient period [36]. To our knowledge, this is the first report characterizing the metabolic profile of the cow throughout gestation.

Urea is a source of non-protein nitrogen that can be used as a protein supplement due to its chemical composition [37]. Preston et al. [38] demonstrated an influence of dietary protein intake on concentrations of nitrogen in the blood. Similar results were found by others [39,40,41,42] who also observed elevated concentrations of PUN in animals fed a diet high in protein. The authors did not report a difference in concentration of PUN between treatments in the current manuscript. However, circulating concentrations of urea are dependent on dietary protein concentration, protein degradability in the rumen, and interval from meal [43]. In the current report, cattle were fed a single meal daily that differed by urea inclusion or exclusion, and blood samples for metabolic analysis were collected after a period of feed withdrawal; to this distinction, one can potentially attribute the differences between the current and prior literature.

While differences were not observed by treatment, the inclusion of the pelleted supplement and progression of gestation influenced circulating concentrations of PUN in the current report. Importantly, protein requirements increase throughout gestation, and diets were adjusted to meet dietary recommendations by stage of gestation. As such, we observed elevated concentrations of PUN in the subsequent blood samples collected at 56 and 224 d of gestation. These dietary-induced alterations do not account for differences observed at 140 and 280 d of gestation. Others have demonstrated that animals adapt to high-protein rations [44], as a result, dietary-induced elevations in PUN are muted when offered regularly as part of the ration. A similar phenomenon may account for the cyclic loss in difference between select samples (84, 112, 168, 196, and 252 d of gestation) collected following the addition of the pelleted supplement to the ration.

Previous research has investigated the effects of protein supplementation on the reproductive and metabolic aspects of gestating heifers [45]. That report demonstrated that protein supplementation affects BW when protein is offered during the last 3 wk of gestation, but protein supplementation—even when extended into lactation—did not affect milk production during the first 8 wk of lactation. Differences in gain of BW in response to supplementation between the aforementioned study and the one presented herein may be attributed to the source, duration, and access to protein supplementation, as well as differences in physiological state and parity. In the current report, BW of cows increased during the second half of gestation. However, BW did not differ between treatments. These results were similar to the ones presented by Wilson et al. [46] that provided 129% of the CP requirement for cows in late gestation. Interestingly, cows in the current dataset lost BW between 252 and 280 d of gestation, irrespective of treatment. This reduction in BW coincided with an increase in concentration of NEFA, suggesting that lipid mobilization occurred in order to respond to elevated nutritional requirements, possibly in preparation for lactation. In response to this increased requirement, mobilization of tissues, especially fat, occurs in an effort to maintain homeostasis [47]. In the current dataset, a reduction in backfat thickness was observed between early and late gestation. However, no differences in backfat thickness were observed between treatments. Similarly, Wilson et al. [46] demonstrated that maternal high protein diets during late gestation failed to alter maternal weight or condition, but progeny of these dams had greater backfat thickness. When synthesis of mammary secretions is initiated during the last 4 to 6 wk of gestation, nutritional requirements of the dam increase. As mammary secretions are generated, the mobilization of energy stores and altered utilization of nutrients becomes necessary [48], potentially contributing to the difference in subcutaneous adiposity between early and late gestation.

In order to prepare for lactation, metabolism changes from an anabolic to a catabolic state. Lipolysis of adipose tissue increases, as observed in the current study, to increase the concentration of NEFA for the production of glucose in the liver. When comparing the mammary secretions collected at hour 0 and hour 24 postpartum, we were able to demonstrate differences in volume, weight, total lactose, percentage of protein, and concentration of milk urea nitrogen. A considerable amount of research has been done in dairy cows comparing composition of mammary secretion at different times after parturition as well as comparing different diets. However, in beef cattle, research has been conducted only comparing different breeds and how milk composition affects calf performance [49,50].

Limited data exists pertaining to mammary secretion quantity or quality in the periparturient period of beef cows. In a similar report with cross-bred beef cows maintained on range, concentrations of milk urea nitrogen were lower than observed in the present study, likely a function of reduced dietary CP content [51]. Moreover, mammary secretions for assessment of quantity and quality in the referenced report were performed following peak lactation (day 70 of lactation), compared to the neonatal period in the current report. Considerable differences exist in the composition of mammary secretions as lactation progresses. Colostrum, the initial high-density product of lactation, is the primary source of Ig and nutrients required for neonatal survival. The low volume, nutrient-dense mammary secretions of the neonatal period align with the limited capacity of the neonatal calf stomach. Moreover, it is necessary that the calf acquire passive transfer of immunity from the dam’s mammary secretions postnatally. In the current report, supplementation of urea provided no benefit in regard to mammary secretions essential for neonatal survival (i.e., Ig, lactose, fat, and protein) of the calf, if allowed to nurse postnatally.

The level of dietary protein offered to dairy cows during late gestation had no effect on milk yield or composition in early lactation [36,44]. Waterman et al. [51] demonstrated that age-matched beef cows grazing forage containing different CP content did not result in differences in milk urea nitrogen content, as observed in the present report. However, Waterman et al. [51] was able to demonstrate an influence of CP content of available forage on milk yield and content. Unfortunately, the aforementioned reference was not able to record feed intake or compare the influence of supplemental urea or protein on mammary secretions. As such, the current report is the first to demonstrate the influence of supplemental urea on mammary secretions in the beef cow.

## 5. Conclusions

We conclude that cows are able to maintain metabolic homeostasis and pregnancy by mobilizing adipose depots in order to maintain a glucose gradient that serves as a fuel reservoir for the offspring in utero, as supported by the lipid analysis data presented in the current work Supplementation of excessive urea does not relieve gestation-induced adipose tissue catabolism of the dam. Furthermore, providing dietary urea to increase the protein available to the dam beyond dietary recommendations throughout gestation had no effect on parameters that directly influence neonatal morbidity and mortality rates. Thus, employment of a nutritional strategy during gestation that targets nutritional recommendations is paramount for the animal’s homeostasis and the maintenance of its metabolic parameters.

## Figures and Tables

**Table 1:** Ingredients and chemical composition of feed offered to cows from day 28 of gestation to parturition.

Item	Control Pellet ^a^	Urea Pellet ^b^	Chopped Forage A ^c^	Chopped Forage B ^c^
Ingredient, % of DM				
Wheat midds	55.54	46.73	-	-
Barley ground	10.00	10.00	-	-
Soybeans hulls	10.00	10.00	-	-
Urea	-	8.81	-	-
Malt sprouts	7.50	7.50	-	-
Calcium	6.90	6.90	-	-
Cane molasses	5.00	5.00	-	-
Bentonite power	4.00	4.00	-	-
Salt	0.68	0.68	-	-
Selenium	0.10	0.10	-	-
Mid-mature grass hay	-	-	100.00	-
Wheat straw	-	-	-	100.00
Analyzed composition, % of DM				
ADF ^d^	11.85	10.91	42.90	48.10
NDF	30.38	27.24	-	-
TDN	60.78	54.61	53.60	47.70
CP	14.38	38.21	13.90	5.68
Calculated composition				
NEm, Mcal/kg	1.43	1.30	1.12	0.97

^a^ The Control Pellet was fed to the Control treatment. ^b^ The Urea Pellet was fed to the Urea treatment. ^c^ Chopped Forage A and B were fed to both treatments with proportions adjusted throughout gestation to meet or exceed dietary recommendations (NASEM, 2016). ^d^ ADF, acid detergent fiber.

**Table 2:** Effect of dietary urea during gestation on beta-hydroxybutyrate (BHB), non-esterified fatty acids (NEFA), glucose, and plasma urea nitrogen (PUN) in multiparous cows. Dietary treatments were: Control (0 g urea/animal/day) or Urea (80 g urea/animal/day). Treatments were offered from day 28 of gestation to parturition.

	Treatment			*p*-Value		
	Control	Urea	SEM	Treatment	Day	Treatment by Day
BHB, mmol/L	1.14	1.22	0.103	0.60	0.003	0.79
NEFA, mmol/L	0.43	0.45	0.018	0.41	<0.0001	0.98
Glucose, mmol/L	3.28	2.93	0.373	0.53	0.04	0.75
PUN, mmol/L	2.98	3.63	0.197	0.06	0.01	0.08

**Table 3:** The effects of dietary urea during gestation on mammary secretion volume, weight, and composition in samples collected at 0 and 24 h after parturition. Dietary treatments were: Control (0 g urea/animal/day) or Urea (80 g urea/animal/day). Treatments were offered from day 28 of gestation to parturition. Total mammary secretion composition was calculated as parameter multiplied by mammary secretion weight (fat, lactose, protein, and solids-not-fat) or volume (Ig, somatic cell count, and urea nitrogen).

	Treatment			Sample			*p*-Value		
	Control	Urea	SEM	Hour 0	Hour 24	SEM	Treatment	Sample	Treatment by Sample
Volume, L	3.79	2.68	0.554	2.54	3.93	0.395	0.21	<0.0001	0.95
Weight, g	3816.25	2707.50	575.710	2557.50	3966.25	410.080	0.22	<0.0001	0.91
Fat, %	7.43	7.18	0.922	7.35	7.25	1.007	0.86	0.95	0.85
Total Fat, g	279.12	199.73	45.262	196.99	281.86	45.262	0.25	0.22	0.70
Ig, mg/mL	93.33	86.67	15.635	90.00	-	11.055	0.78	-	-
Total Ig, g	220.80	189.60	26.335	205.20	-	18.622	0.45	-	-
Lactose, %	3.12	3.10	0.178	2.67	3.56	0.386	0.88	0.11	0.19
Total Lactose, g	117.62	88.25	16.014	64.57	141.30	16.014	0.23	0.01	0.58
Protein, %	10.73	12.07	0.746	14.71	8.09	1.485	0.20	0.05	0.82
Total Protein, g	398.49	304.12	76.288	373.28	329.33	76.288	0.37	0.64	0.70
Solids-not-fat, %	24.39	24.83	0.970	27.61	21.62	1.936	0.74	0.15	0.36
Total Solids-not-fat, g	917.96	673.00	131.980	729.88	861.09	131.980	0.23	0.54	0.63
Somatic Cell Count, cells*1000/L	1924.43	3114.29	1177.330	1638.97	3399.75	1948.670	0.23	0.35	0.32
Total Somatic Cell Count, cells*1000	5224.41	8867.89	3381.670	3488.20	10,604.00	5867.250	0.25	0.25	0.26
Urea Nitrogen, mg/dL	26.20	34.96	2.250	38.76	22.40	3.813	0.02	0.05	0.18
Total Urea Nitrogen, g	0.98	0.89	0.205	0.97	0.90	0.205	0.72	0.79	0.73

## Data Availability

Data will be made available, upon reasonable request to the corresponding author.
